# Molecular Mechanism of Action of Trimethylangelicin Derivatives as CFTR Modulators

**DOI:** 10.3389/fphar.2018.00719

**Published:** 2018-07-04

**Authors:** Onofrio Laselva, Giovanni Marzaro, Christian Vaccarin, Ilaria Lampronti, Anna Tamanini, Giuseppe Lippi, Roberto Gambari, Giulio Cabrini, Christine E. Bear, Adriana Chilin, Maria C. Dechecchi

**Affiliations:** ^1^Program in Molecular Medicine, Hospital for Sick Children, Toronto, ON, Canada; ^2^Department of Pharmaceutical and Pharmacological Sciences, University of Padova, Padova, Italy; ^3^Department of Life Sciences and Biotechnology, University of Ferrara, Ferrara, Italy; ^4^Laboratory of Molecular Pathology, Department of Pathology and Diagnostics, University Hospital of Verona, Verona, Italy; ^5^Department of Biochemistry, University of Toronto, Toronto, ON, Canada; ^6^Department of Physiology, University of Toronto, Toronto, ON, Canada

**Keywords:** trimethylangelicin, cystic fibrosis, cystic fibrosis transmembrane conductance regulator, correctors, potentiators

## Abstract

The psoralen-related compound, 4,6,4′-trimethylangelicin (TMA) potentiates the cAMP/PKA-dependent activation of WT-CFTR and rescues F508del-CFTR-dependent chloride secretion in both primary and secondary airway cells homozygous for the F508del mutation. We recently demonstrated that TMA, like lumacaftor (VX-809), stabilizes the first membrane-spanning domain (MSD1) and enhances the interface between NBD1 and ICL4 (MSD2). TMA also demonstrated anti-inflammatory properties, via reduction of IL-8 expression, thus making TMA a promising agent for treatment of cystic fibrosis. Unfortunately, TMA was also found to display potential phototoxicity and mutagenicity, despite the fact that photo-reactivity is absent when the compound is not directly irradiated with UVA light. Due to concerns about these toxic effects, new TMA analogs, characterized by identical or better activity profiles and minimized or reduced side effects, were synthesized by modifying specific structural features on the TMA scaffold, thus generating compounds with no mutagenicity and phototoxicity. Among these compounds, we found TMA analogs which maintained the potentiation activity of CFTR in FRT-YFP-G551D cells. Nanomolar concentrations of these analogs significantly rescued F508del CFTR-dependent chloride efflux in FRT-YFP-F508del, HEK-293 and CF bronchial epithelial cells. We then investigated the ability of TMA analogs to enhance the stable expression of varying CFTR truncation mutants in HEK-293 cells, with the aim of studying the mechanism of their corrector activity. Not surprisingly, MSD1 was the smallest domain stabilized by TMA analogs, as previously observed for TMA. Moreover, we found that TMA analogs were not effective on F508del-CFTR protein which was already stabilized by a second-site mutation at the NBD1-ICL4 interface. Altogether, our findings demonstrate that these TMA analogs mediate correction by modifying MSD1 and indirectly stabilizing the interface between NBD1 and CL4.

## Introduction

Cystic fibrosis (CF) is an autosomal recessive genetic disease caused by mutations in the cystic fibrosis transmembrane conductance regulator (*CFTR*) gene. Over 2,000 sequence variations have been reported so far, many of which have been defined as causative (see the Cystic Fibrosis Mutation Database of the Cystic Fibrosis Gene Analysis Consortium^1^) (Riordan et al., 1989; Gadsby et al., 2006). CFTR is an ABC transporter, containing two membrane-spanning domains (MSD1 and MSD2), with a total of four intracellular loops (ICL1-4), two nucleotide-binding domains (NBD1 and NBD2), and a regulatory domain (R) (Riordan, 2008). CFTR activation requires ATP binding to the interface between NBD1 and NBD2, in addition to protein kinase A (PKA)-mediated phosphorylation of the R domain (Cheng et al., 1991; Berger et al., 2005). CFTR acts as a chloride channel expressed on the apical membrane of epithelial cells, where its function is essential for maintenance of salt and fluid homeostasis. The deletion of phenylalanine at position 508 (F508del), the most common disease-causing CFTR mutation, is located in NBD1 and exists in ∼90% of patient alleles (Veit et al., 2016; Mijnders et al., 2017). F508del is a class II CF mutation, which results in a folding defect in the full-length F508del-CFTR, which is mediated through the NBD1:ICL4 interface. Improper folding leads to premature degradation by the ubiquitin-proteasome system. This degradation, due to lack of cell surface CFTR expression, then causes ionic imbalance and aberrant fluid homeostasis at epithelial surfaces in the lung, thus producing thick mucus and causing impairment of mucociliary clearance, ultimately predisposing the patients to bacterial infections, inflammation and impaired lung function (Boucher, 2007).

Small-molecule compounds, which are known to modulate either defective F508del-CFTR folding and processing (correctors) or channel gating (potentiators), are promising therapeutic strategies for treatment of CF by targeting the underlying defects in CFTR protein (Pedemonte et al., 2005). CFTR correctors increase the cell-surface expression of F508del-CFTR either indirectly, by acting as proteostasis regulators and thus modulating the activity of proteins involved in folding, degradation or trafficking of F508del-CFTR (Hanrahan et al., 2013), or directly, through binding as pharmacological chaperones to F508del-CFTR (Okiyoneda et al., 2013). Unlike conventional correctors needing several hours to effectively rescue protein expression, potentiators interact directly with CFTR, to rapidly increase CFTR channel activation by either ATP-dependent (Hwang and Sheppard, 2009) or ATP-independent (Eckford et al., 2012) mechanisms.

KALYDECO^TM^, developed by Vertex Pharmaceuticals and also known as ivacaftor (VX-770), is a small-size potentiator approved for treatment of patients carrying the CFTR class III mutation G551D, along with nine other rare ‘gating’ defect mutations (Van Goor et al., 2009, 2014; Yu et al., 2012). The two additional correctors lumacaftor (VX-809) and Tezacaftor (VX-661), also developed by Vertex Pharmaceuticals, promote protein stability and forward trafficking of F508del-CFTR to cell surface (Van Goor et al., 2009, 2011; Hanrahan et al., 2017). ORKAMBI^TM^ has recently emerged as the first combination therapy. In combination with ivacaftor, lumacaftor was found to significantly enhance the functional activity of F508del-CFTR in pre-clinical studies of primary bronchial cells and rectal biopsy-derived organoids (Van Goor et al., 2011; Dekkers et al., 2013).

The mechanism of action of lumacaftor (recently categorized as a Class I corrector) has been investigated by several groups (Loo et al., 2013; Ren et al., 2013; Laselva et al., 2016, 2018; Hudson et al., 2017; Loo and Clarke, 2017). Previous studies using isolated domains of CFTR showed that the N-terminal domain of CFTR (i.e., MSD1) was important for the stabilizing effect of lumacaftor (Loo et al., 2013; Ren et al., 2013; Laselva et al., 2016). In the context of the full-length mutant protein F508del-CFTR, a secondary “rescue” mutation that partially compensates for defective interaction of NBD1 and ICL4, namely R1070W, partially abrogated the correction mediated by lumacaftor; thus arguing that lumacaftor mediates its correction via allosteric effects on this interface (He et al., 2013; Okiyoneda et al., 2013; Laselva et al., 2018). As yet, it is not clear how interaction of lumacaftor at MSD1 mediates such changes in inter-domain interaction. Okiyoneda et al. (2013) proposed a scheme wherein lumacaftor and other correctors that act via this mechanism are described as Class I correctors.

It has been more recently reported that prolonged treatment with the potentiator ivacaftor induces a dose-dependent reversal of the lumacaftor-mediated F508del-CFTR rescue in human bronchial epithelial cultures. An increased turnover rate at the cell surface of rescued F508del-CFTR, and the resulting decreased stability, have been proposed as the underlying mechanisms (Cholon et al., 2014; Veit et al., 2014).

These findings suggest that use of compounds with dual activity (i.e., corrector and potentiator) may be an appealing therapeutic perspective for CF treatment.

Previous studies showed that the psoralen-related compound 4,6,4′-trimethylangelicin (TMA) potentiates cAMP/PKA-dependent activation of WT-CFTR (Tamanini et al., 2011) and rescues F508del-CFTR-dependent chloride secretion in both primary and secondary airway cells homozygous for the F508del mutation (Favia et al., 2014; Abbattiscianni et al., 2016). We recently demonstrated that TMA, like lumacaftor, stabilizes the first membrane-spanning domain (MSD1) of CFTR (Laselva et al., 2016) and enhances the interface between NBD1 and ICL4 (MSD2) (Laselva et al., 2018) The inhibitory effect on *Pseudomonas aeruginosa*-dependent IL-8 transcription at nanomolar concentrations in CF-derived bronchial epithelial cells (Tamanini et al., 2011) is another interesting property of TMA. Therefore, TMA appears to act as a triple-acting compound, exhibiting anti-inflammatory activity, in addition to corrector and potentiator properties. TMA was designated by EMA as an Orphan Drug for the treatment of cystic fibrosis with the code EU/3/13/1137.

Despite these promising properties, TMA has some potential drawbacks, such as mutagenicity (Bianchi et al., 1990) and photo-reactivity toward DNA pyrimidine bases (Bordin et al., 1991), albeit the latter is not observed when the compound is not directly irradiated with UVA light.

Due to our expertise in the field of furocoumarin derivatives with anti-inflammatory properties (Borgatti et al., 2011; Marzaro et al., 2013, 2015), we designed a small library of TMA analogs to help determine whether different properties of TMA are dependent upon specific functional groups present on the scaffold of parent compound, and to identify which structural determinants possess biological activity (Supplementary Figure S1). Furthermore, we aimed to establish whether the side effects could be reduced or even abolished through specific structural modifications, which do not impair the biological properties of TMA.

The first series of derivatives showed good anti-inflammatory properties, via NF-κB inhibition (Lampronti et al., 2017), and paved the way to identifying two interesting compounds, 4,7,4′-trimethylallopsoralen (ALLO) and 4,6-dimethylangelicin (DMA) (Supplementary Figure S1), which deserve further investigation into their mechanism of action, although these two isomers showed residual photoactivity and mutagenicity.

A second series was designed and synthesized, in an effort to abolish or minimize these side effects (Marzaro et al., 2018). Three additional non-mutagenic compounds were subsequently be identified: 4-isopropyl-6-ethyl-4′-methylangelicin (IPEMA), 4-propyl-6-ethyl-4′-methylangelicin (PEMA) and 4-isopropyl-6,4′-dimethylangelicin (IPDMA) (Supplementary Figure S1). Interestingly, IPEMA lacks photoreactivity, whilst PEMA and IPDMA have some residual photoreactive potential under direct UVA irradiation.

Therefore, the aim of this study was to explore the effect of TMA analogs on CFTR function and determine their mechanism of action.

## Materials and Methods

### Compounds

lumacaftor and ivacaftor were obtained by Selleckchem (United States), TMA and TMA analogs were partly synthesized as previously described (Guiotto et al., 1984, 1995; Marzaro et al., 2018) and partly belong to the collection of the Organic Synthesis Lab (Department of Pharmaceutical Sciences, University of Padova). DMA was newly synthesized as described in the Supplementary Material. TMA and TMA analogs were dissolved in DMSO at a final concentration of 200 μM (stock solution), whereas lumacaftor and ivacaftor were dissolved in DMSO at a final concentration of 10 mM.

### Cell Lines

Fischer Rat Thyroid (FRT) epithelial cells, stably co-expressing human F508del (FRT-F508del) or G551D (FRT-G551D) CFTR and the high-sensitivity halide-sensing green fluorescent analog yellow fluorescent protein (HS-YFP) YFP-H148Q/I152L were a generous gift from L. J. Galietta (Telethon Institute of Genetics and Medicine, Pozzuoli, Italy). Cells were grown in Coon’s Modified Ham’s F-12 media plus 10% fetal bovine serum (FBS), L-glutamine, and penicillin/streptomycin at 37°C under 5% CO_2_ (Pedemonte et al., 2010). CuFi-1 cells, a generous gift of A. Klingelhutz, P. Karp, and J. Zabner (University of Iowa, Iowa City), is a human bronchial epithelial cell line derived from a CF patient (CuFi-1, F508del/F508del CFTR mutant genotype) and transformed by reverse transcriptase component of telomerase (hTERT), human papillomavirus type 16 (HPV-16) E6 and E7 genes (Zabner et al., 2003). These cells were grown on human placental collagen type VI (Sigma, St. Louis, MO, United States) coated flasks in BEGM medium (Cambrex Bio Science Walkersville, Walkersville, MD, United States), as described (Zabner et al., 2003).

CFBE41o- cells, stably overexpressing F508del-CFTR (CFBE41o-F508del) homozygous for the F508del allele (F508del/F508del) (a generous gift from J. P. Clancy, University of Cincinnati, Children’s Hospital Medical Center, Cincinnati, OH, United States) are human bronchial epithelial cells, grown in complete media (EMEM, 10% FBS, L-glutamine, and penicillin/streptomycin) in the presence of 2 μg/ml puromycin positive selection. Cells were routinely grown on flasks coated with an extracellular matrix containing fibronectin/vitrogen/BSA, at 37°C under 5% CO_2_.

Human Embryonic Kidney (HEK)-293 GripTite cells (HEK) from Dr. Daniela Rotin (Hospital for Sick Children, Toronto, ON, Canada) were maintained in DMEM (Wisent, St-Bruno, QC, Canada) supplemented with non-essential amino acids (Life Technologies, Waltham, MA, United States) and 10% FBS (at 37°C with 5% CO_2_ (HEPA incubator, Thermo Electron Corporation) and processed as previously described (D’Antonio et al., 2013; Molinski et al., 2015). Transient transfections were performed using PolyFect Transfection Reagent (Qiagen, Hilden, Germany), according to the manufacturer’s protocol, as previously described (Molinski et al., 2015; Laselva et al., 2016).

### Fluorescence Measurements of Iodide Influx

Iodide influx was measured in FRT-YFP F508del cells, as described elsewhere (Pedemonte et al., 2005) from the kinetics of YFP fluorescence decrease following addition of extracellular iodide in the presence of CFTR stimulating cocktail. Cells were seeded on round glass coverslips and pre-incubated for 48 h with vehicle, TMA, TMA analogs or lumacaftor. At the time of assay, cells were washed in Dulbecco’s PBS (in mM: 137 NaCl, 2.7 KCl, 8.1 Na_2_HPO_4_, 1.5 KH_2_PO_4_, 1 CaCl_2_, and 0.5 MgCl_2_, pH 7.4) and subsequently incubated with stimulation cocktail (20 μM forskolin and 5 μM ivacaftor) in presence or absence of 10 μM CFTR inhibitor, CFTRInh-172 (Sigma), for 30 min. FRT-G551D cells were washed as indicated above and incubated with stimulation cocktail (20 μM forskolin and vehicle or genistein, ivacaftor, TMA or TMA analogs) in the presence or absence of CFTRInh-172 for 30 min. The cover slips were then transferred onto a Nikon TMD inverted microscope, through a Nikon Fluor 40 objective, and signal was acquired with a Hamamatsu C2400–97 charge-coupled intensified video camera at a rate of 1 frame/3 s. Fluorescence coming from each single cell of at least five cells per field was analyzed by a customized software (Spin, Vicenza, Italy). Results are presented as transformed data to obtain the percentage signal variation [ΔF(t)] relative to the time of addition of stimulus, according to the equation: ΔF(t) = 100[F(t) – F(0)]/F(0), where Ft and F0 are the fluorescence values at the time t, and at the time of iodide addition, respectively. YFP fluorescence decay rate was calculated by fitting fluorescence data of time courses by means of an exponential function. CFTR activity was calculated as the difference between YFP fluorescence decay rate in absence or in the presence of CFTRInh-172. The assay of each sample consisted of continuous 120-s fluorescence readings with injection, 40 s before and 80 s after, of iodide-rich Dulbecco’s PBS (in mM: 137 NaI, 2.7 KCl, 8.1 Na_2_HPO_4_, 1.5 KH_2_PO_4_, 1 CaCl_2_, and 0.5 MgCl_2_, pH 7.4), to reach a final iodide concentration of 50 mM, as described (Favia et al., 2014).

### CFTR-Dependent Chloride Efflux in Bronchial Epithelial Cells

Cystic fibrosis transmembrane conductance regulator function was assessed in CuFi-1 and CFBE41o-F508del cells by single-cell fluorescence imaging, using the potential-sensitive probe DiSBAC2(3) (Molecular Probes, Eugene, OR, United States) as previously reported (Dechecchi et al., 2007). CFTR dependent Cl^-^ channel activity was stimulated by a cAMP elevating cocktail consisting of 20 μM forskolin plus 5 μM ivacaftor. CFTRInh-172 was added to a final concentration of 10 μM. Briefly, cells grown on coated round glass coverslips were washed in a Cl^-^ containing solution (mM): 101 Na^++^, 114 Cl^-^, 5 K^+^, 2 Ca^2+^, 2 Mg^2+^, 50 mannitol, 5 glucose, 5 HEPES-Tris, pH 7.4, mounted in a perfusion chamber (Medical System, Baltimore, MD, United States), and perfused for 10–15 min at 25°C with a Cl^-^ free solution (mM): 101 Na^+^, 106 gluconate, 14 acetate, 5 K^+^, 2 Ca^2+^, 2 Mg^2+^, 50 mannitol, 5 glucose, 5 HEPES-Tris, pH 7.4, containing 100 nM DiSBAC2(3). The time courses were performed at 25°C and a baseline signal was acquired before the addition of the stimulus. Results are presented as transformed data to obtain the percentage signal variation [ΔF(t)] relative to the time of stimulus addition according to the equation: ΔF(t) = 100[F(t)–F(0)]/F(0), where Ft and F0 are the fluorescence values at the time t and at the time of stimulus addition, respectively.

### CFTR Channel Function Using Membrane Depolarization Assay

Cystic fibrosis transmembrane conductance regulator functional measurements were performed with fluorometric imaging plate reader (FLIPR) membrane depolarization assay in HEK-293 cells, using the blue membrane potential dye (Molecular Devices, San Jose, CA, United States) which can detect changes in transmembrane potential.

HEK-293 cells stably overexpressing F508del-CFTR or HEK-293 cells transiently transfected with F508del/R1070W construct, were grown to 100% confluence in 96-well plates (black, flat bottom; Costar) and treated for 24 h with 3 μM VX-809, 200 nM TMA, 200 nM IPEMA, 200 nM IPDMA, 200 nM DMA, 200 nM ALLO or DMSO. Cells were washed with PBS and the blue membrane potential dye, dissolved in chloride-free buffer containing 136 mM sodium gluconate, 3 mM potassium gluconate, 10 mM glucose, 20 mM HEPES, pH 7.35, 300 mOsm, at a concentration of 0.5 mg/ml, was added to the cells for 1 h at 37°C (Laselva et al., 2016; Ahmadi et al., 2017; Molinski et al., 2017). The plate was then read in a fluorescence plate reader (SpectraMax i3; Molecular Devices) at 37°C. After reading baseline fluorescence, CFTR was stimulated with the cAMP agonist forskolin (10 μM; Sigma-Aldrich) and the potentiator ivacaftor (1 μM; Selleck Chemicals). CFTR-mediated depolarization of membrane was detected as an increase in fluorescence, whilst repolarization or hyperpolarization was detected as a decrease (Maitra et al., 2013; Ahmadi et al., 2017). To terminate the assay, CFTR-specific inhibitor 172 (CFTRinh-172, 10 μM; Cystic Fibrosis Foundation Therapeutics) was added to all wells. Changes in membrane potential were normalized to fluorescence before addition of agonist (forskolin).

### Western Blotting

HEK-293 expressing F508del-CFTR or transiently transfected with F508del/R1070W-CFTR, were grown to 100% confluence in 24-well plates (Costar) and treated for 24 h with 3 μM lumacaftor, 200 nM TMA, 200 nM IPEMA, 200 nM IPDMA, 200 nM DMA, 200 nM ALLO or DMSO. Cells were then lyzed in modified radioimmunoprecipitation assay buffer (50 mM Tris-HCl, 150 mM NaCl, 1 mM EDTA, pH 7.4, 0.2% SDS, and 0.1% Triton X-100) containing a protease inhibitor cocktail (Roche) for 10 min, and the soluble fractions were analyzed by sodium dodecyl sulfate-polyacrylamide gel electrophoresis (SDS-PAGE) on 6% Tris-Glycine gels (Life Technologies). After electrophoresis, proteins were transferred to nitrocellulose membranes (Bio-Rad) and incubated in 5% milk. CFTR bands were detected with human CFTR specific murine mAb 596 (1:10,000; University of North Carolina at Chapel Hill). Relative expression level of CFTR proteins were quantified by densitometry of immunoblots, using ImageJ software version 1.46 (National Institutes of Health).

### CFTR Steady-State Levels

Steady-state levels of CFTR truncation fragments were assayed with Western Blot analysis. HEK cells were transiently transfected with ΔNBD2 (containing residues 1–1172), ΔR (700–835 del) MSD1-NBD1-R (containing residues 1–845), MSD1 (containing 1–380), MSD1-D373X (containing residues 1–372) and MSD2 (containing residues 837–1196). Transient transfections were performed using PolyFect Transfection Reagent (Qiagen, Hilden, Germany), according to the manufacturer’s protocol, as previously described (Molinski et al., 2015; Laselva et al., 2016, 2018). After 18 h of transfection, the cells were treated with 3 μM lumacaftor, 200 nM TMA, 200 nM IPEMA, 200 nM IPDMA, 200 nM DMA, 200 nM ALLO or vehicle control (DMSO) and 24 h after correction the cells were lyzed in modified radioimmunoprecipitation assay buffer (50 mM Tris-HCl, 150 mM NaCl, 1 mM EDTA, pH 7.4, 0.2% SDS, and 0.1% Triton X-100) containing a protease inhibitor cocktail (Roche) for 10 min. The soluble fractions were then analyzed by sodium dodecyl sulfate-polyacrylamide gel electrophoresis (SDS-PAGE) on 6% (ΔNBD2 and ΔR) or 4–12% Tris-Glycine gels (Life Technologies). After electrophoresis, proteins were transferred to nitrocellulose membranes (Bio-Rad) and incubated in 5% milk. CFTR bands then were detected with human CFTR-MSD1-specific murine mAb MM13-4 (1:10,000; EMD Millipore, Billerica, MA, United States) or with monoclonal antibody A52 for MSD2 (1:1,000) (Dr. David Clarke, University of Toronto) (Loo et al., 2013; Laselva et al., 2016).

### Statistics

Statistical analysis was carried out with non-parametric Mann–Whitney *U* test and, one-way ANOVA, using Prism 7.0 Software (GraphPad Software, San Diego, CA, United States). One-way/two-way analyses of variance were conducted, when appropriate (^∗^*P* < 0.05, ^∗∗^*P* < 0.01, ^∗∗∗^*P* < 0.001, and ^∗∗∗∗^*P* < 0.0001).

## Results

### Effect of TMA Analogs on FRT-YFP-G551D Cells

**FIGURE 1:**
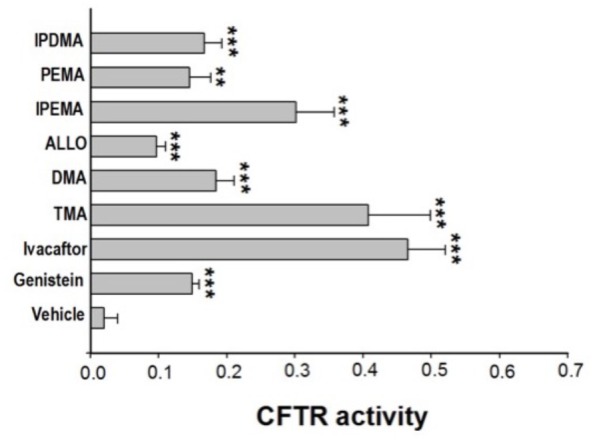
Effect of TMA analogs on CFTR-dependent chloride efflux in FRT-G551D cells. FRT-YFP-G551D cells, grown on round glass coverslips, were treated for 30 min with vehicle alone or genistein (50 μM), ivacaftor (5 μM), TMA or its analogs (200 nM), before the fluorescence assay. CFTR-dependent chloride efflux was assayed by single-cell fluorescence imaging analysis of YFP fluorescence quenching by iodide, stimulated by forskolin (20 μM), in the presence or absence of CFTRInh-172 (10 μM). The assay of each sample consisted of a continuous 120-s fluorescence reading with 40 s before and 80 s after injection of the iodide-rich Dulbecco’s PBS. Results are presented as transformed data, to obtain the percentage signal variation [ΔF(t)] relative to the time of addition of iodide, according to the equation: ΔF(t) = 100[F(t)–F(0)]/F(0), where Ft and F0 are the fluorescence values at the time t and at the time of addition of iodide, respectively. YFP fluorescence decay rate was calculated by fitting with an exponential function fluorescence data of time course. CFTR activity was obtained by the difference between YFP fluorescence decay rate in absence and in the presence of CFTRInh-172. Fluorescence coming from each single cell of at least five cells per field was recorded. Each bar corresponds to the mean ± SEM of data points coming from at least three different experiments. Statistical comparisons were made using non-parametric Mann–Whitney *U* test (^∗^*P* < 0.05, ^∗∗^*P <* 0.01, and ^∗∗∗^ *P* < 0.001).

Some linear and angular furocoumarins, such as 5- or 8-MOP and angelicin, have been shown to act as potentiators of CFTR-mediated chloride transport (Devor et al., 1997). We recently found that also TMA potentiates the cAMP/PKA-dependent activation of wild-type or surface expressed F508del-CFTR (Tamanini et al., 2011). Therefore, the effect of acute incubation with these new TMA analogs on class III CFTR mutations was tested in FRT-YFP-G551D cells, in comparison with genistein, ivacaftor, and TMA. As reported in **Figure 1**, the analogs DMA, ALLO, IPEMA, PEMA, and IPDMA significantly potentiated G551D-CFTR-dependent chloride activity. DMA, ALLO, PEMA, and IPDMA were comparable to genistein. IPEMA was the most effective, although its efficacy was lower than that of TMA or ivacaftor. These results indicate that the structural modifications on the drug scaffold maintain TMA’s potentiating activity of CFTR.

### Effect of TMA Analogs on FRT-YFP-F508del Cells

It was previously shown that TMA rescues surface expression and channel function of F508del-CFTR in primary and secondary human bronchial epithelial cells (Favia et al., 2014).

**FIGURE 2:**
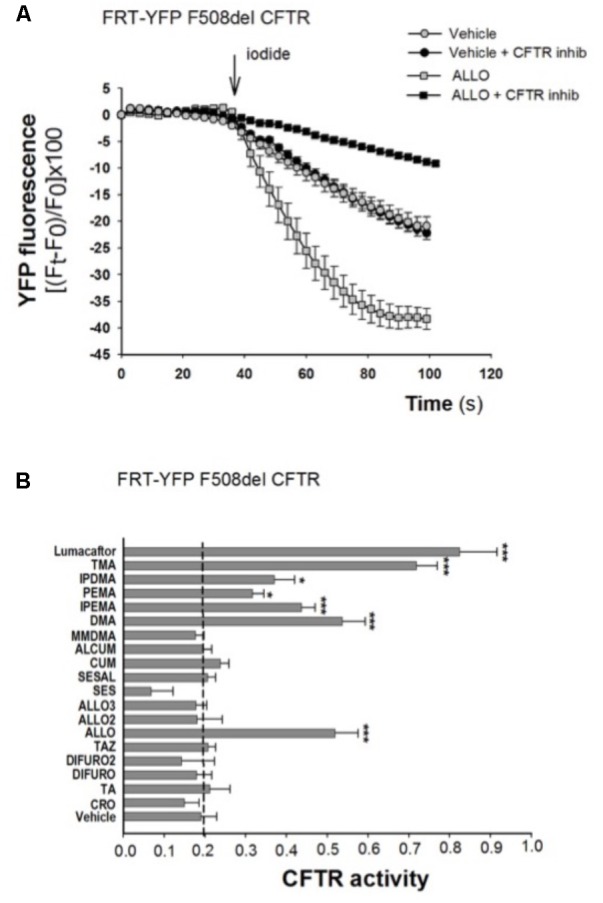
Effect of TMA analogs on CFTR-dependent chloride efflux in FRT-F508del cells. FRT-YFP-F508del cells, grown on round glass coverslip, were treated for 48 h with vehicle alone or lumacaftor (5 μM), TMA or its analogs (200 nM). CFTR function was assayed at 48 h as indicated in the **Figure 1** legend, in the presence of forskolin (20 μM) plus ivacaftor (5 μM). **(A)** Representative traces showing iodide influx in control conditions (vehicle) or after 48 h incubation with ALLO. **(B)** Summary of data from different TMA analogs compared to TMA and lumacaftor. CFTR activity was calculated as indicated in **Figure 1** legend. Each bar represents the mean ± SEM of at least three different experiments. Dashed line corresponds to cells treated with vehicle. Statistical comparisons were made using non-parametric Mann–Whitney *U* test (^∗^*P* < 0.05, ^∗∗^*P <* 0.01, and ^∗∗∗^*P* < 0.001).

To explore the role of these analogs as correctors of F508del-CFTR, we first performed the analysis of CFTR-mediated chloride transport in the model of FRT-YFP-F508del cells, after long-term incubation with different compounds. TMA and TMA analogs (200 nM), lumacaftor -809 (5 μM) or vehicle were added to FRT-YFP-F508del cells for 48 h at 37°C, and correction of F508del-CFTR function was assessed by measuring the decrease of YFP fluorescence upon addition of extracellular iodide and with the activating cocktail (20 μM forskolin, and 5 μM ivacaftor). As shown by a representative experiment, correction of F508del-CFTR resulted in larger reduction of YFP fluorescence induced by increased iodide influx compared to cells treated with vehicle alone. The CFTR-dependent decrease of YFP-fluorescence signal was verified by traces obtained in the presence of CFTRInh-172 (**Figure 2A**). The TMA analogs (Supplementary Figure S1) were compared to TMA and lumacaftor. **Figure 2B** shows that among these compounds, five TMA analogs (DMA, ALLO, IPEMA, PEMA, and IPDMA) significantly rescued F508del-CFTR-dependent activity in FRT-YFP-F508del cells, after 48 h pre-incubation at 37°C. In particular, DMA, ALLO, IPEMA, and IPDMA were the most effective correctors of F508del-CFTR, although to a lesser extent than TMA or lumacaftor.

We hence selected these TMA analogs and performed dose-response experiments, in order to evaluate their potency and efficacy. Cells were treated for 48 h at 37°C with vehicle alone, or different concentrations (50–500 nM) of TMA analogs. Values of CFTR activity in cells treated with different concentrations of TMA analogs were fitted by a rectangular hyperbola and EC_50_ and maximum effect were calculated.

As shown in **Table 1**, DMA, ALLO, IPEMA, and IPDMA were very potent correctors, being EC_50_ values in the nanomolar range. IPEMA and DMA were more powerful than ALLO and IPDMA. Among these analogs, IPDMA was the most effective, since CFTR activity was about fivefold higher than that of untreated cells.

**Table 1:** Potency and efficacy of TMA analogs as correctors of F508del CFTR in FRT-YFP F508del cells.

TMA analog	EC_50_ (nM)	CI (nM)	Maximum effect (CFTR activity)	CI (CFTR activity)
ALLO	156	76	0.626	0.11
DMA	55	31	0.448	0.05
IPEMA	40	20	0.523	0.06
IPDMA	164	83	1.434	0.32

*FRT-YFP F508del cells were treated with increasing concentrations from 50 to 500 nM of selected TMA analogs: ALLO, DMA, IPEMA, and IPDMA. CFTR activity was measured as indicated in “Materials and Methods” section. Data obtained from at least three different experiments were fitted by a rectangular hyperbola with *Graph Pad prism 6.0* in order to obtain potency (EC_50_) and efficacy (Maximum effect). CI = 95% confidence interval.*

### Effect of TMA Analogs in Combination With TMA or Lumacaftor on FRT-YFP F508del Cells

**FIGURE 3:**
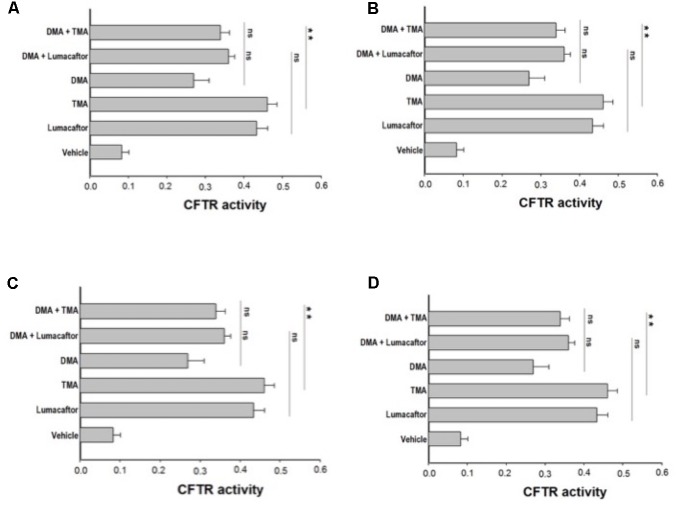
Effect of treatment combinations in rescuing F508del CFTR function in FRT-F508del cells. FRT-YFP-F508del cells, grown on round glass coverslips, were treated for 48 h with vehicle alone or lumacaftor (5 μM), TMA (200 nM), DMA (100 nM) **(A)**, ALLO (100 nM) **(B)**, IPEMA (100 nM) **(C)**, IPDMA (100 nM) **(D)** alone, or in combination with TMA or lumacaftor. CFTR-dependent chloride efflux was assayed as indicated in **Figure 1** legend. CFTR activity was calculated as indicated in **Figure 1** legend. Each bar corresponds to the mean ± SEM of at least three different experiments. Statistical comparisons were made using non-parametric Mann–Whitney *U* test (^∗^*P* < 0.05, ^∗∗^*P <* 0.01, and ^∗∗∗^*P* < 0.001).

Ideally, the combination of a potentiator with multiple correctors, each with a different mechanism of action should be seen as an especially promising approach, since it should promote further improvement in drug efficacy and clinical benefit for CF patients. In order to evaluate a possible additive effect of TMA analogs with correctors lumacaftor and TMA, we treated FRT-YFP F508del cells with TMA analogs DMA, ALLO, IPEMA, and IPDMA (100 nM) alone or in combination with TMA or lumacaftor (**Figures 3A–D**). None of these TMA analogs improved the correcting effect of lumacaftor or TMA (**Figure 3**), thus suggesting that lumacaftor, TMA and TMA analogs share a similar mechanism of action related to the rescue F508del-CFTR function. These results correlate with data previously observed for TMA and lumacaftor (Favia et al., 2014; Laselva et al., 2016). Notably, the combination of TMA with the structurally related analogs DMA or ALLO (**Figures 3A,B**), significantly reduced the CFTR activity compared to TMA alone.

### Effect of TMA Analogs on CF Human Bronchial Epithelial Cells

**FIGURE 4:**
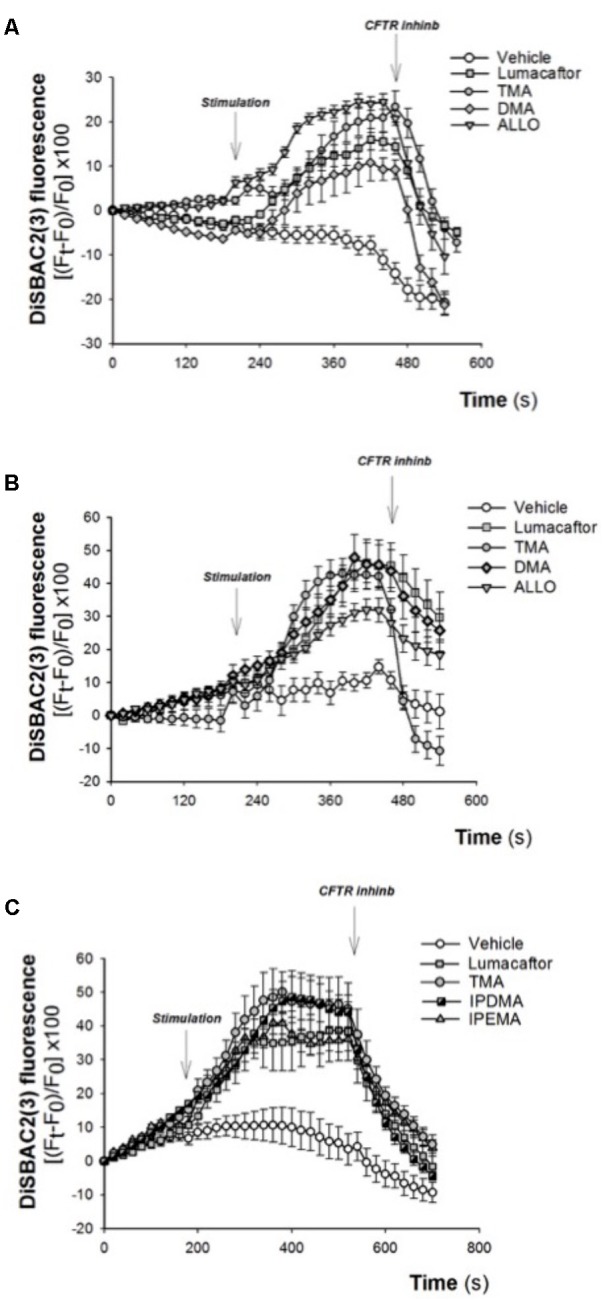
Effect of selected TMA analogs DMA, ALLO, IPEMA, and IPDMA on CFTR-dependent chloride efflux in CF human bronchial cells. CFTR-dependent chloride function was assayed by single cell fluorescence imaging, using a membrane-potential sensitive dye. **(A)** CuFi-1 cells grown on round glass coverslips were treated for 48 h with vehicle or lumacaftor (5 μM), TMA (200 nM) or DMA (200 nM) or ALLO (200 nM) and then mounted on the perfusion chamber and perfused with Cl^-^ free solution containing DiSBAC2(3) to allow the equilibration of the dye within the cell membrane. The arrows indicate the time of the addition of the stimulus: forskolin (20 μM) and ivacaftor (5 μM) or the CFTRInh-172 (10 μM). Fluorescence signal from each single cell of at least five cells per field was analyzed and represented by time course tracings. Data represent the mean ± SEM of the relative fluorescence collected from all the cells in the field. A representative of four independent experiments is shown. **(B)** CFBE41o-F508del cells, were treated as indicated in **(A)**. A representative of three independent experiments is shown. **(C)** CFBE41o-F508del cells were treated for 48 h with vehicle or lumacaftor (5 μM), TMA (200 nM) or IPDMA (200 nM) or IPEMA (200 nM). A representative of four independent experiments is shown.

The corrector activity was earlier found to be strongly dependent on cell background, with the extreme case of many compounds working only on a single cell type (Pedemonte et al., 2010). TMA rescued F508del-CFTR-dependent chloride secretion and cell surface expression in both primary and secondary airway cell monolayers homozygous for F508del mutation (Favia et al., 2014), thus indicating that the correcting effect of TMA is not influenced by cell background. The effect of TMA analogs was tested in CF human bronchial epithelial cells CuFi-1 (**Figure 4A**) and CFBE41o-F508del (**Figures 4B,C**), incubated for 48 h at 37°C in the presence of vehicle alone or lumacaftor or TMA or analogs. Data were then analyzed by single cell digital imaging using the membrane potential-sensitive fluorescence probe DiSBAC2(3) (Dechecchi et al., 2007). As shown in **Figure 4**, activation of CFTR increased the DiSBAC2(3) fluorescence in CF cells treated with correctors, as a function associated with cell membrane depolarization in the presence of a Cl^-^ gradient. The DiSBAC2(3) fluorescence decrease obtained by CFTRInh-172 further confirms the specificity of this CFTR-mediated chloride efflux measured in this functional assay. Similar to lumacaftor and TMA, TMA analogs DMA, ALLO (**Figures 4A,B**), IPEMA, and IPDMA (**Figure 4C**) rescued F508del-CFTR function in CF human bronchial epithelial cells.

### The Corrector Activity of TMA Analogs in HEK-293 Cells

Previous studies showed that both lumacaftor and TMA partially rescued the processing defect of F508del-CFTR, such that the immature core-glycosylated (Band B) form of the protein both acquired complex-glycosylation (Band C in SDS-PAGE) and exhibited partial function at the plasma membrane (Van Goor et al., 2009; Favia et al., 2014). We recently demonstrated that both modulators rescued F508del-CFTR through stabilization of the first membrane spanning domain (MSD1) (Laselva et al., 2016). We now were capable to identify the mechanism of action of these novel TMA analogs, as previously described.

**FIGURE 5:**
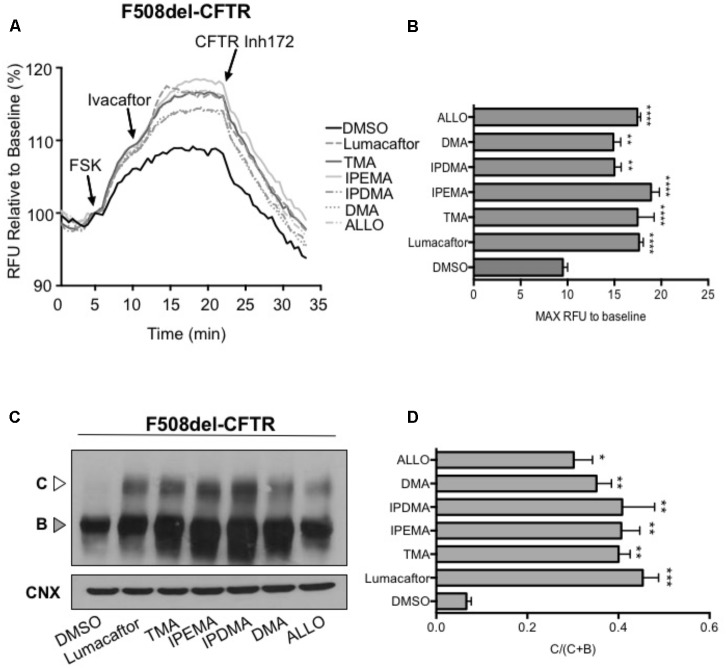
TMA analogs rescued F508del-CFTR similarly to lumacaftor and TMA. **(A)** Functional analysis in HEK cells stably transfected with F508del-CFTR, using the fluorometric imaging plate reader membrane depolarization assay, in the presence or absence of correctors. Following 5 min baseline measurement, CFTR was activated by forskolin (FSK) (1 μM). After 5 min incubation, a CFTR potentiator (ivacaftor, 1 μM) was added for 10 min. CFTR inhibitor (CFTRinh-172, 10 μM) was then added to deactivate CFTR. **(B)** Shows the mean (±SEM) of maximal activation of CFTR after stimulation by FSK and ivacaftor (*n* = 3). Statistical comparisons were made using one-way ANOVA test (^∗∗^*P* < 0.01 and ^∗∗∗∗^*P <* 0.0001). **(C)** HEK F508del-CFTR stable transfected cells were treated in the presence or absence of correctors for 24 h at 37°C. C, mature complex-glycosylated CFTR; B, immature core-glycosylated CFTR. **(D)** Bar graphs show the mean (±SEM) of ratio C/(C + B) of CFTR (*n* = 3). Statistical comparisons were made using one-way ANOVA test (^∗^*P* < 0.05, ^∗∗^*P <* 0.01, and ^∗∗∗^*P <* 0.001).

We first performed functional measurements using the FLIPR membrane depolarization assay in HEK-293 cells (Laselva et al., 2016; Ahmadi et al., 2017), showing that TMA analogs (200 nM) rescued F508del-CFTR-dependent chloride conductance in HEK-293 cells incubated for 24 h at 37°C (**Figures 5A,B**). These TMA analogs were also capable to increase abundance of both the immature (Band B) and mature bands (Band C) of F508del-CFTR (**Figures 5C,D**).

### TMA Analogs Stabilize N-Terminal CFTR Fragments That Contain MSD1

**FIGURE 6:**
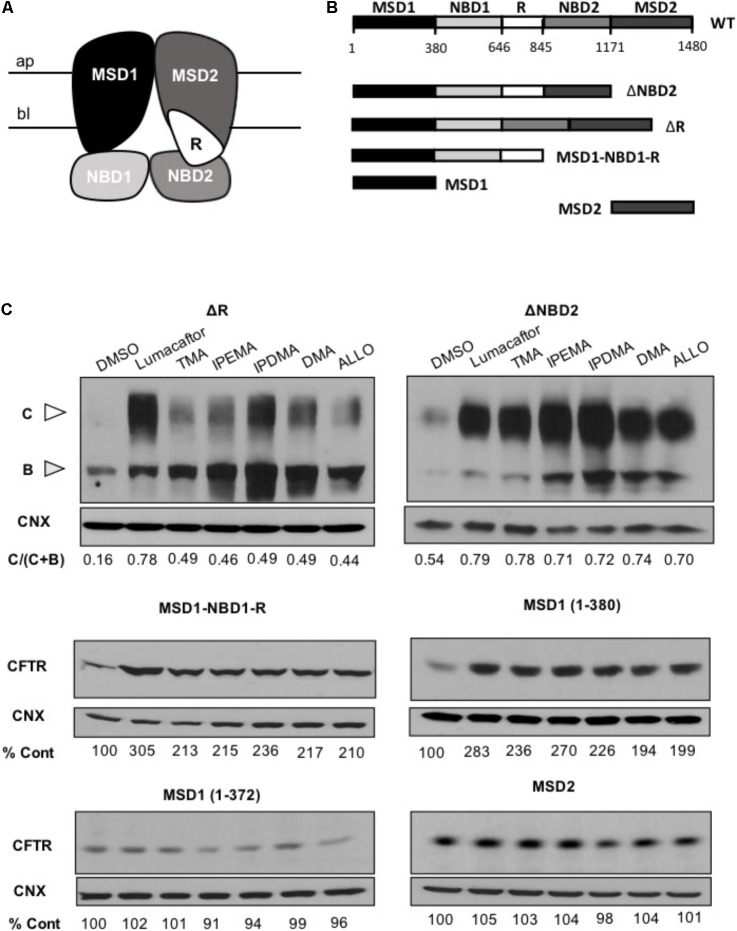
TMA analogs stabilize MSD1 of CFTR. **(A)** Graphical image of the CFTR structure. **(B)** Domain boundaries of CFTR fragments used in this study. **(C)** The effect of lumacaftor (3 μM), TMA (200 nM), DMA (200 nM), ALLO (200 nM), IPEMA (200 nM), and IPDMA (200 nM) on the abundance of CFTR fragments. Data are representative of three experiments. % abundance of CFTR was normalized firstly to calnexin loading and then to DMSO control.

To identify the region(s) of CFTR required for corrector activity of these TMA analogs, we then expressed different lengths CFTR fragments in HEK-293 cells (**Figure 6**).

Cells were treated for 24 h at 37°C with 3 μM lumacaftor, 200 nM TMA, or 200 nM TMA analogs (IPEMA, IPDMA, DMA, ALLO). Like what has been observed with lumacaftor and TMA, pre-incubation with all TMA analogs promoted enhanced steady state levels of all CFTR fragments including MSD1 residues (1–380). TMA analogs did not increase the steady-state abundance of MSD2 (**Figure 6C**), thus suggesting that these TMA analogs interact with and stabilize the MSD1.

### Stabilization of NBD1/ICL4 Interface in F508del Is Modified Correction by TMA Analogs

Substitution of native arginine with a tryptophan at position 1070 in the F508del-CFTR protein (i.e., R1070W in ICL4) was shown to partially repair the assembly defect in the full-length mutant protein, thus suggesting that assembly of CFTR can be partially restored through structural changes at relevant interfaces even in the absence of residue F508 (Pedemonte et al., 2005; Mendoza et al., 2012; Molinski et al., 2012).

**FIGURE 7:**
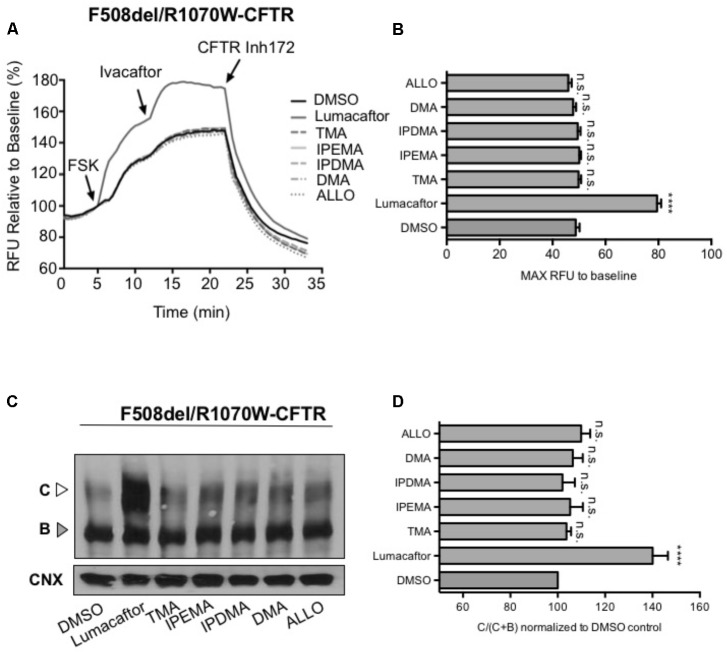
TMA and its analogs failed to have an additive effect with the second-site mutation R1070W. **(A)** Functional measurements, using membrane depolarization dye assay, of F508del/R1070W-CFTR. HEK293 cells were transiently transfected with F508del/R1070W-CFTR mutant and treated for 24 h with: DMSO, lumacaftor (3 μM), TMA (200 nM), DMA (200 nM), ALLO (200 nM), IPEMA (200 nM), and IPDMA (200 nM). Following 5 min baseline measurements, CFTR was activated by FSK (10 μM). After 5 min incubation, ivacaftor (1 μM) was acutely added, and after 10 min, the CFTR inhibitor (CFTRinh-172, 10 μM) was added to deactivate CFTR. **(B)** Bar graph represents the mean (±SEM) of maximum chloride efflux rates after CFTR activation of three experiments. Statistical comparisons were made using one-way ANOVA test (^∗∗∗∗^*P* < 0.0001). **(C)** HEK293 cells were transiently transfected with the F508del/R1070W-CFTR mutant and treated for 24 h at 37°C with DMSO, lumacaftor (3 μM), TMA (200 nM), DMA (200 nM), ALLO (200 nM), IPEMA (200 nM), and IPDMA (200 nM). C: mature, complex-glycosylated CFTR; B: immature, core-glycosylated CFTR. **(D)** Bars represent the mean (±SEM) of the ratio of CFTR bands [C/(C+B)] of three experiments. Statistical comparisons were made using one-way ANOVA test (^∗∗∗∗^*P* < 0.0001).

To assess the effect of TMA analogs on the F508del-CFTR protein, which was already stabilized by a second-site mutation at the NBD1-ICL4 interface, we expressed F508del/R1070W in HEK-293 cells and studied the effect of TMA analogs on F508del/R1070W-dependent chloride efflux. In agreement with previous data (Okiyoneda et al., 2013), lumacaftor significantly enhanced F508del/R1070W-dependent chloride efflux and protein expression (**Figures 7A,C**), whilst both TMA and its analogs were ineffective (**Figures 7B,D**).

## Discussion

A large number of pharmacological modulators have been identified, which can rescue the expression and/or function of the mutated CFTR protein. The potentiator ivacaftor alone, or in combination with the corrector lumacaftor, are the only pharmacological modulators of CFTR currently approved for treatment of CF. Nevertheless, lumacaftor therapy in combination with ivacaftor does not seem to provide a significant improvement in the lung function of F508del homozygous patients. It has also recently been reported that long-term ivacaftor treatment enhances the turnover of rescued F508del-CFTR at the cell surface, and induces a significant decrease of cell surface stability (Cholon et al., 2014; Veit et al., 2014), thus suggesting that identification of alternative dual-acting CFTR modulators may be a valuable perspective. Dual-acting small-molecules have been recently identified, which independently promote F508del-CFTR trafficking to the plasma membrane and boost its channel activity (Pedemonte et al., 2011; Phuan et al., 2011; Liu et al., 2018).

In the present study, we describe dual acting TMA analogs which are effective in both correcting F508del-CFTR and potentiating CFTR-dependent chloride transport. This evidence supports previous data obtained using TMA (Favia et al., 2014), thus indicating that structural modifications of TMA scaffold maintain its ability to modulate CFTR activity. More specifically, the insertion of more hindered substituents at the fourth position of the angelicin scaffold, as IPEMA, preserves the CFTR correction and potentiation properties of this compound, and allows for the prevention of unwanted photoreactivity and mutagenicity, which characterized the parent TMA molecule. Interestingly, IPEMA is the most effective potentiator and the most potent corrector among these TMA analogs, which makes it a promising agent for the treatment of CF. TMA exhibits independent corrector and potentiator activities, similar to those of lumacaftor and ivacaftor. Like lumacaftor, it also interacts directly with MSD1 of CFTR (Laselva et al., 2016). Our data demonstrates that these independent corrector and potentiator activities are preserved in the structurally related TMA analogs.

In FRT cells, 100 nM TMA induced an increase of CFTR activity similar to that produced by 5 mM lumacaftor, supporting data previously reported (Favia et al., 2014) (**Figures 2B**, **3**). Although TMA analogs herein described are very potent F508del CFTR correctors, their efficacy seems to be lower than that of TMA or lumacaftor (**Figures 2B**, **3**).

Rescue of F508del-CFTR function by TMA analogs IPEMA, IPDMA, DMA, ALLO was observed in FRT and HEK-293 cells, and also in human bronchial epithelial cells CuFi-1 and CFBE41o-F508del. This indicates that the corrector effect is not dependent on cell background and that these analogs also share similar mechanisms as TMA, since simultaneous addition of TMA and its analogs fails to elicit an additive effect on the F508del-CFTR processing defect.

Similar to lumacaftor and TMA, functional rescue of F508del-CFTR by TMA analogs was accompanied by a significant increase of the fully glycosylated mature Band C, representing F508del-CFTR protein which as trafficked from the ER to Golgi and plasma membrane. When we compared the efficacy of TMA analogs with that observed for lumacaftor or TMA, the steady state levels of the mature Band C were found to be very similar. Interestingly, both TMA and its structurally related analogs exhibit greater potency in enhancing steady state abundance of Band C compared to lumacaftor. Notably, two TMA analogs, IPEMA and IPDMA, considerably increased immature Band B expression of F508del-CFTR, thus suggesting that these compounds may increase the stability of the F508del-CFTR immature form, at an early phase of its biogenesis.

In the present study, we hence demonstrate that TMA and its analogs rescue F508del-CFTR by stabilization of the NBD1/ICL4 interface. Using the R1070W second-site/suppressor mutation, which enhances stability of the NBD1/ICL4 interdomain interface, in the context of F508del, we also observed that TMA and its analogs failed to show an additional effect on relative abundance of Band C. These findings are contradictory to those of lumacaftor, for which additional correction of the processing defect was observed in the context of the double mutant. Therefore, lumacaftor corrects F508del-CFTR by modifying multiple intramolecular interactions in addition to F508del-NBD1 and ICL4.

The TMA analogs described in this study exhibited several advantages which would make them promising therapeutic agents in CF. They were found to be dual correctors and potentiators, acting through binding to the MSD1 domain of CFTR, stabilizing the interface between NBD1 and ICL4, facilitating correct folding and ultimately preventing premature degradation. According to previous evidence of anti-inflammatory properties exerted through inhibition of NF-κB/DNA interactions, along with lower risk of side effects compared to the parent TMA, some TMA analogs such as DMA, ALLO, and IPEMA should hence be further investigated in view of their possible role for treating CF. Therefore, these TMA analogs may be seen as a promising therapeutic option for CF disease due to the absence or minimized mutagenicity and photo-reactivity potential.

## Author Contributions

MD, AC, CB, GC, and RG planned the study. MD, GC, OL, and CB designed the experiments and analyzed the data. GM and CV synthesized TMA analogs under supervision by AC. MD and GC supervised the experiments on FRT and CF bronchial cells. OL performed the experiments on HEK-293 cells. IL, AT, GL, and RG critically revised the manuscript. MD, OL, AC, GC, and CB wrote the manuscript.

## Conflict of Interest Statement

The authors declare that the research was conducted in the absence of any commercial or financial relationships that could be construed as a potential conflict of interest.

## Footnotes

1 www.genet.sickkids.on.ca/cftr/
