# Supplementary material for: Molecular Mechanism of Action of Trimethylangelicin Derivatives as CFTR Modulators
Source: Front Pharmacol. 2018 Jul 4;9:719. doi: 10.3389/fphar.2018.00719 (PMC6039571; doi:10.3389/fphar.2018.00719)
Supplement: Supplementary file 1 [file Presentation_1.PDF]

Supplementary Material

Molecular mechanism of action of Trimethylangelicin derivatives as CFTR modulators

Onofrio Laselva[1§], Giovanni Marzaro[2§], Christian Vaccarin[2], Ilaria Lampronti[3], Anna Tamanini[4], Giuseppe Lippi[4], Roberto Gambari[3], Giulio Cabrini [4], Christine Bear[1,5,6#], Adriana Chilin[2#], Maria Cristina Dechecchi[4#]*

[1]*Programme in Molecular Medicine, Hospital for Sick Children, Toronto, Canada*
[2]*Department of Pharmaceutical and Pharmacological Sciences - University of Padova, Padova, Italy*
[3]*Department of Life Sciences and Biotechnology - University of Ferrara, Ferrara, Italy*
[4] *Laboratory of Molecular Pathology, Department of Pathology and Diagnostics-University Hospital of Verona, Verona, Italy*
[5] *Department of Biochemistry, University of Toronto, Canada*
[6] *Department of Physiology, University of Toronto, Canada*

§ Onofrio Laselva and Giovanni Marzaro contributed equally to this work.

Christine Bear, Adriana Chilin and Maria Cristina Dechecchi share senior authorship

*** Correspondence:** cristina.dechecchi@aovr.veneto.it

Contents **pag**

Supplementary Figures: Figure S1 **S2**

Supplementary Data: synthesis of DMA **S3**

^{13}C-NMR and HRMS spectra of DMA **S5**

Supplementary Figures: Figure S1

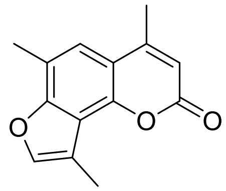
TMA

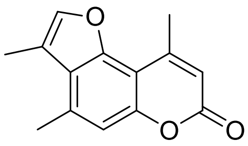
ALLO

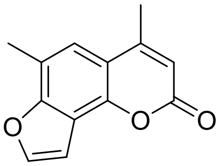
DMA

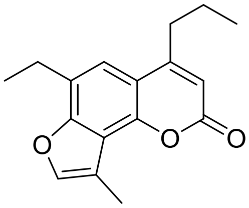
PEMA

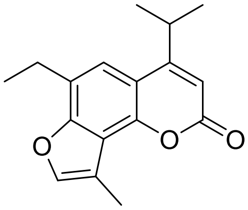
IPEMA

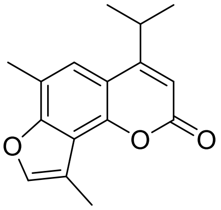
IPDMA

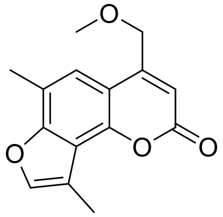
MMDMA

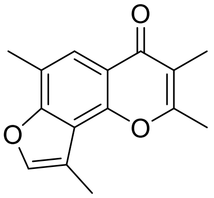
CRO

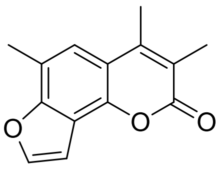
TA

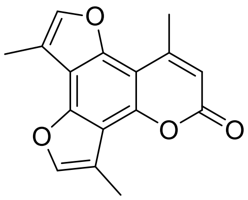
DIFURO

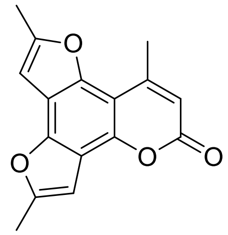
DIFURO2

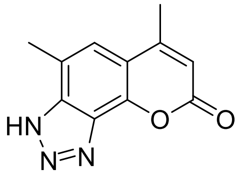
TZA

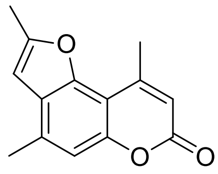
ALLO2

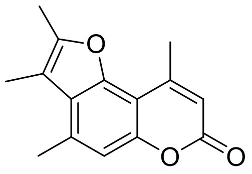
ALLO3

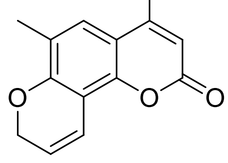
SES

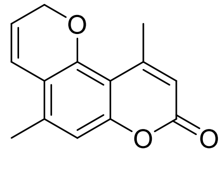
SESAL

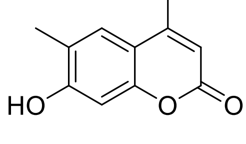
CUM

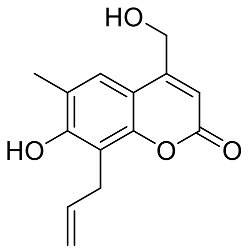
ALCUM

Figure S1. Structures of the tested compounds.

Supplementary Data: Synthesis of DMA

General Information. All commercial chemicals and solvents used were analytical grade and were used without further purification. Microwave assisted reactions were performed on a CEM Discover monomode reactor in closed devices with the temperature monitored by a built-in infrared sensor and the automatic control of the power. Melting points (uncorrected) were determined using a Gallenkamp MFB-595-010M capillary melting point apparatus. Analytical thin layer chromatography (tlc) was performed on pre-coated silica gel plates (Merck 60-F-254, 0.25 mm), which were developed on a mixture of $CHCl_3$/MeOH (9/1). The NMR spectra were recorded on a Bruker 300-AMX spectrometer with TMS as internal standard. Coupling constants are given in Hz. HRMS spectra were acquired using a XEVO G2-S Qtof (Waters) mass spectrometer with direct injection of the sample and collecting data in positive ion mode.

Synthesis of 2,4-dihydroxytoluene

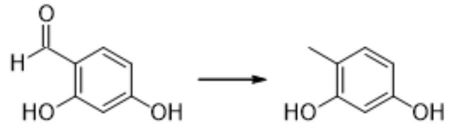

To a solution of dihydroxybenzaldheyde (1.4 g, 10.0 mmol) in tetrahydrofuran (40 mL) sodium cyanoborohydride (2.0 g, 3.2 mmol) was added portionwise, then HCl 1M (40 mL) was added dropwise. The mixture was stirred at room temperature for 3 h. The solution was diluted with water (50 mL) and extracted with Et_2O (3 x 50 mL). The organic phase was evaporated under reduced pressure and the residue was purified by column chromatography, eluting with $CHCl_3$, to give 2,4-dihydroxytoluene (1.2 g, yield 96%); mp 104 °C. ^{1}H-NMR (DMSO-*d6*): 9.02 (broad s, 1H, 2-OH or 4-OH), 8.89 (broad s, 1H, 2-OH or 4-OH), 6.77 (d, *J* = 8.3 Hz, 1H, 6-H), 6.24 (d, *J* = 2.4 Hz, 1H, 3-H), 6.09 (dd, *J* = 8.3 and 2.4 Hz, 1H, 5-H), 1.97 (s, 3H, 1-Me).

Synthesis of 4,6-dimethyl-7-hydroxybenzopyran-2-one

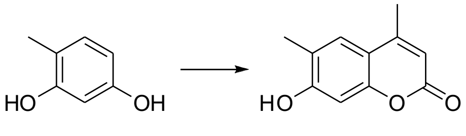

To a solution of 2,4-dihydroxytoluene (1.1 g, 9.0 mmol) in ethyl acetoacetate (1.2 g, 1.1 mL, 9.0 mmol), H_2SO_4 (7 mL) was added dropwise and the mixture was stirred at room temperature for 1 h. The solution was poured into an ice/water mixture (100 g) and the obtained precipitate was filtered

and washed with water to give the corresponding 4,6-dimethyl-7-hydroxybenzopyran-2-one (1.1 g, yield 64%), mp 254 °C. ^{1}H-NMR (CD_3OD): 7.61 (s, 1H, 5-H), 6.58 (s, 1H, 8-H), 6.21 (q, *J* = 1.1 Hz, 1H, 3-H), 2.57 (d, *J* = 1.1 Hz, 3H, 4-Me), 2.39 (s, 3H, 6-Me).

Synthesis of 4,6-dimethyl-7-(2’,2’-diethoxyethoxy)benzopyran-2-one

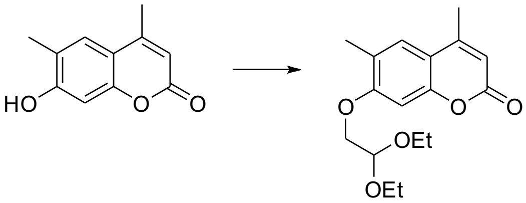

A mixture of 4,6-dimethyl-7-hydroxybenzopyran-2-one (1.0 g, 5.3 mmol), chloroacetaldehyde diethylacetale (1.0 g, 1.0 mL, 6.7 mmol) and anhydrous K_2CO_3 (2.0 g) in DMF (20 mL) was refluxed 6 h. The mixture was poured into water (100 mL) and the obtained precipitate was filtered and washed with water to give 4,6-dimethyl-7-(2’-diethoxyethoxy)benzopyran-2-one (0.8 g, yield 50%), mp 78 °C. ^{1}H-NMR ($CDCl_3$): 7.31 (s, 1H, 5-H), 6.76 (s, 1H, 8-H), 6.12 (q, *J* = 1.1 Hz, 1H, 3-H), 4.88 (t, *J* = 5.2 Hz, 1H, -OC$\underline{H}$-CH_2O), 4.05 (t, *J* = 5.2 Hz, 2H, -OCH-C$\underline{H}_2$O), 3.79 and 3.66 (dq, *J* = 9.4 and 7.0 Hz, 2H each, -OC$\underline{H}_2$-CH_3), 2.39 (d, *J* = 1.1 Hz, 3H, 4-Me), 2.27 (s, 3H, 6-Me), 1.26 (t, *J* = 7.0 Hz, 6H, -OCH_2-C$\underline{H}_3$).

Synthesis of 4,6-dimethyl-2*H*-furo[2,3-*h*]-1-benzopyran-2-one

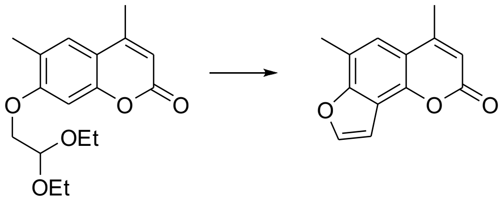

A solution of 4,6-dimethyl-7-(2’-diethoxy-ethoxy)benzopyran-2-one (0.5 g, 1.6 mmol) in TFA (5 mL) was microwave irradiated at 120 °C (power set point 200W; ramp time 1 min; hold time 5 min). The reaction mixture was poured into water (100 mL) and the obtained precipitate was filtered off. The solid was purified by column chromatography, eluting with $CHCl_3$, to give 4,6-dimethyl-2*H*-furo[2,3-*h*]-1-benzopyran-2-one (0.2 g, yield 58%), mp 246 °C. ^{1}H-NMR ($CDCl_3$): 7.69 (d, *J* = 2.2 Hz, 1H, 8-H), 7.29 (s, 1H, 5-H), 7.14 (d, *J* = 2.2 Hz, 1H, 9-H), 6.25 (q, *J* = 1.1 Hz, 1H, 3-H), 2.58 (s, 3H, 6-Me), 2.49 (d, *J* = 1.1 Hz, 3H, 4-Me). ^{13}C-NMR ($CDCl_3$): 161.12, 156.24, 153.54, 146.39, 145.45, 120.41, 118.63, 116.28, 114.55, 112.73, 104.61, 77.20, 19.45, 14.89. HRMS (ESI-TOF) for $C_{13}H_{11}O_3$ [M + H]$^+$: calcd.: 215.0708, found: 215.0765.

^{13}C-NMR and HRMS spectra of DMA

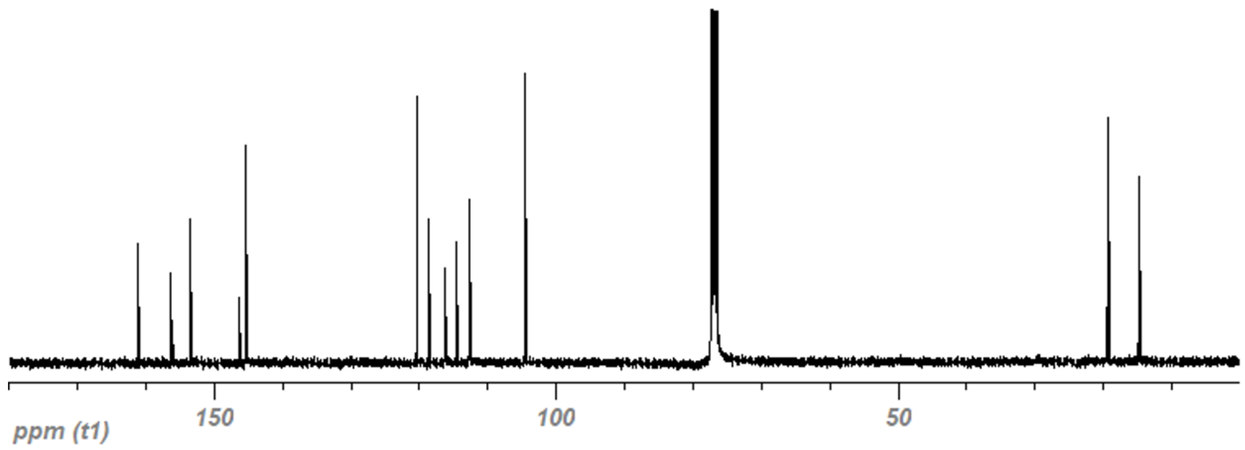

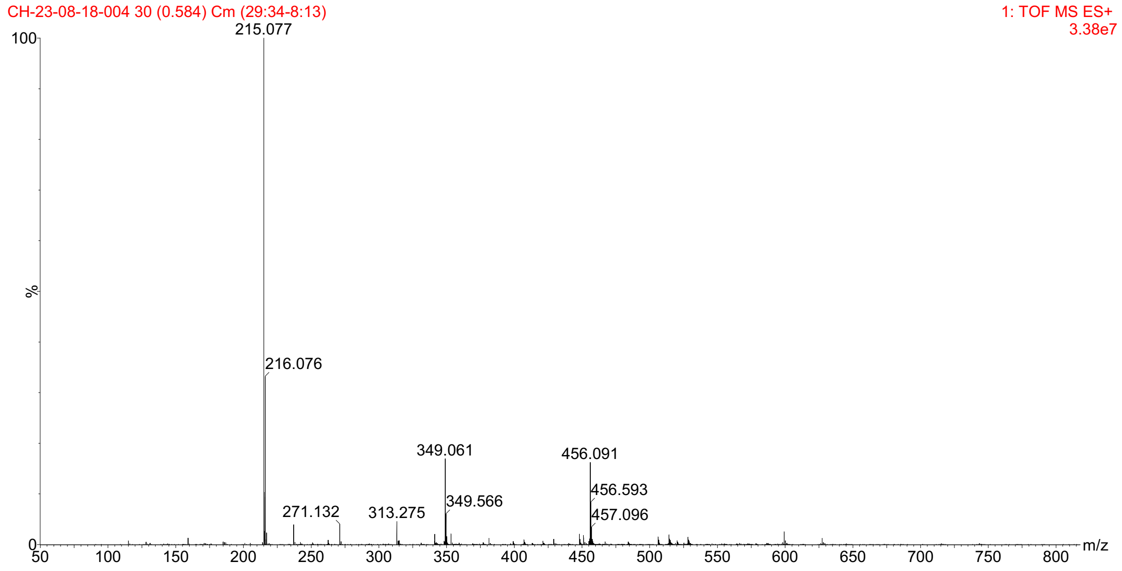

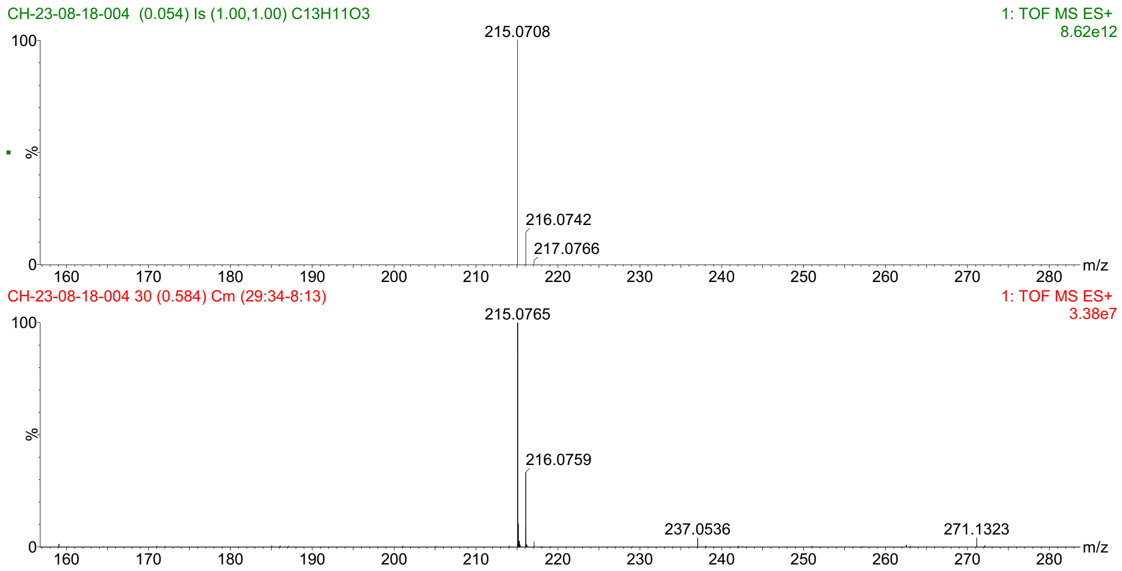
CH-23-08-18-004 (0.054) Is (1.00,1.00) C13H11O3
1: TOF MS ES+
8.62e12
215.0708
216.0742
217.0766
CH-23-08-18-004 30 (0.584) Cm (29:34-8:13)
1: TOF MS ES+
3.38e7
215.0765
216.0759
237.0536
271.1323
%
m/z
